# Associations between plasma biomarkers and changes in cognitive function over two years in people with and without HIV

**DOI:** 10.1097/QAD.0000000000004435

**Published:** 2026-01-14

**Authors:** Jasmini Alagaratnam, Nicholas Bakewell, Ken M. Kunisaki, Patrick W.G. Mallon, Fiona Burns, Laura Waters, Frank A. Post, Memory Sachikonye, Nicki Doyle, Jaime H. Vera, Alan Winston, Caroline A. Sabin

**Affiliations:** a Department of HIV Medicine & Sexual Health, Chelsea & Westminster Hospital NHS Foundation Trust; b Section of Virology, Department of Infectious Disease, Imperial College London; c Institute for Global Health, University College London, London, UK; d Boise VA Medical Center, Boise, USA; e Centre for Experimental Pathogen Host Research, School of Medicine, University College Dublin, Ireland; f Royal Free London NHS Foundation Trust; g Department of Sexual Health and HIV, Central and North West London NHS Trust; h King's College Hospital NHS Foundation Trust; i UK Community Advisory Board (UK-CAB), London; j Department of Global Health and Infection, Brighton and Sussex Medical School, University of Sussex; k Department of Genitourinary Medicine & HIV, St Mary's Hospital, Imperial College Healthcare NHS Trust, London, UK.

**Keywords:** antiretroviral therapy, cognition, HIV, innate immunity, inflammation

## Abstract

**Objective:**

Chronic inflammation may be associated with cognitive disorders in people with HIV on antiretroviral treatment (ART). We examine associations between cognitive function and plasma biomarkers measured in people with HIV and demographically-similar people without HIV in the POPPY study.

**Design:**

Prospective longitudinal cohort study.

**Methods:**

At baseline and 2-year follow-up, participants completed a cognitive test battery. Global T-scores were derived by averaging domain T-scores. We used linear regression to explore associations between changes in Global T-scores and log-transformed plasma biomarkers of neuronal injury, systemic inflammation and innate immune activation. We explored whether effects of biomarkers differed by HIV status.

**Results:**

A total of 349 participants were included (73% people with HIV, median [interquartile range, IQR] age 54 [50–60] years, 85% male, 95% white). Among people with HIV, 98% were on ART, 93% had HIV-RNA ≤50 copies/ml and median [IQR] CD4^+^ cell count was 627 [490 792] cells/mm^3^. Mean [standard deviation (SD)] baseline Global T-score was 47.7 (5.9) which increased to 48.9 (5.5) after a median [IQR] follow-up of 26 [24,29] months. Lower average increases in Global T-scores were seen in those with higher MIP-1α (parameter estimate: −0.27 [95% CI: –0.51,–0.03]/10% increase) and sCD14 (−0.17 [–0.30,–0.03]), though only MIP-1α (−0.46 [–0.58,–0.10]) remained significant after adjustment. There was no evidence that the associations differed by HIV status.

**Conclusion:**

Higher MIP-1α and sCD14 showed small associations with lower average increases in Global T-scores, with no differences by HIV status or inflammatory clusters, highlighting the multifactorial influences on cognitive trajectories in people ageing with and without HIV.

## Introduction

Emerging evidence demonstrates that individuals on effective antiretroviral treatment (ART) continue to exhibit an elevated risk of developing non-AIDS and noninfectious complications compared to age-matched control populations [1–4]. This phenomenon is hypothesized to be partly driven by persistent immune dysfunction, chronic immune activation, and systemic inflammation despite virological suppression in individuals receiving ART [5–7], as evidenced by persistently elevated biomarkers of immune activation and inflammation, compared to populations without HIV [8,9].

HIV-associated dementia is now rarely seen in people taking ART, however milder forms of cognitive disorders continue to be reported in people with HIV on virologically suppressive modern ART [10,11]. The mechanisms underlying the milder forms of cognitive impairment remain unclear. Dysregulation of innate immune responses and chronic inflammation associated with persistently elevated type I and type II interferons, ongoing low-level viral replication, vascular injury, metabolic abnormalities, glial cell dysfunction, decreased white matter integrity and the adverse effects of antiretroviral agents may contribute to the pathogenesis [12–16]. Ongoing progressive neuronal loss has been observed in individuals with HIV on virologically suppressive ART [17], suggesting that neuronal injury may result not only directly from the virus itself, but also from chronic immune activation and inflammation [18]. Furthermore, neurotoxicity may result directly from viral proteins or from certain drugs capable of crossing the blood-brain barrier [19].

While several biomarkers of immune activation [e.g. CCL2, IL-6, soluble CD14 (sCD14), neopterin, kynurenine, quinolinic acid] were associated with more severe forms of cognitive impairment in the pre-ART era [20–25], evidence regarding specific biomarkers that can reliably predict and monitor cognitive function in people on virologically suppressive ART remains limited.

Here, we examine the associations between biomarkers of systemic inflammation and innate immune activation and changes in cognitive function among people with HIV and demographically similar people without HIV.

## Methods

### Study design and participants

The Pharmacokinetic and clinical Observations in PeoPle over fiftY (POPPY) study is a prospective cohort study of people with and without HIV in the United Kingdom (UK) and Ireland. Three sub-cohorts of participants were recruited from eight HIV clinics: 699 people with HIV aged ≥50 years who were of white or black African race, and acquired HIV through sexual routes; 374 people with HIV aged <50 years, who were frequency-matched to the older group of people with HIV on gender, race, sexual orientation and clinic; and 304 HIV-negative controls aged ≥50 years, who were frequency-matched to the older group of people with HIV on age, gender, race, sexual orientation and location (in or out of London). The baseline visit took place between April 2013–January 2016 and the wave 3 POPPY follow-up visit took place between May 2015–February 2018. Further detail on the POPPY study has been described previously [26].

This present analysis uses a subset of POPPY study participants who were part of the POPPY-Sleep sub-study (participants attended one study visit between March 2017 and July 2018) [27], who had valid data on 31 plasma biomarkers analysed using two platforms that are immunoassays based on Enzyme Linked Immunosorbent Assay (ELISA) as part of this sub-study, and who had an assessment of cognitive function at both the baseline and wave 3 POPPY study visits [28]. The inflammatory pathways and plasma biomarkers measured were as follows: coagulation (sCD40L, D-dimer, sP-Selectin), neuronal injury (NFL, S100B), endothelial function (sICAM-1, E-Selectin, sVCAM-1, vWF), systemic inflammation (hsCRP, IL-2, IL-6, TNF-α, IFN-γ, IL-1β, TNF RI, TNF RII), innate immune activation (sCD14, MCP-1, sCD163, MIP-1α, IL-10), immune regulation (IL-1RA, PDL-1, IL-4), microbial translocation (IL-18, I-FABP, IL-12, LBP) and atherosclerosis (Lp-PLA2, MPO) [28].

The derivation and characteristics of the inflammatory plasma biomarker clusters have been previously described [28]. In summary, the clusters were derived by conducting principal components analysis (PCA) on the correlation matrix of 31 log-transformed plasma biomarker concentrations followed by agglomerative hierarchical cluster analysis [28]. Three distinct clusters were identified: gut/immune activation, neurovascular and reference.

### Cognitive function/performance outcomes

Cognitive function was assessed at baseline and wave 3 for the main POPPY study using the Cogstate battery [29]. The battery covered six cognitive domains commonly affected by HIV-associated cognitive impairment: visual learning, psychomotor function, visual attention, executive function, verbal learning, and working memory/attention. T-scores [mean: 50, standard deviation (SD): 10] were derived, and a Global T-score of overall cognitive function was obtained by averaging the domain T-scores [29,30].

The primary outcome of interest was the change in Global T-scores between the two POPPY study visits, with a positive change reflecting an improvement in Global T-scores at the second study visit compared to the first, and a negative change a deterioration.

### Primary and secondary exposures: plasma biomarkers and inflammatory plasma biomarker clusters

For the primary analyses, we examined the associations between the change in Global T-scores between the two POPPY study visits and specific plasma biomarkers selected a priori based on our hypothesis that they would predict changes in cognitive function: NFL, S100B, sCD14, IL-10, MCP-1, sCD163, MIP-1α, IL-2, IL-6 and TNF-α. For the secondary analyses, we examined associations between the change in Global T-scores between the two POPPY study visits by inflammatory plasma biomarker clusters.

### Statistical analyses

Prior to analyses, we excluded participants without data on the primary outcome (change in Global T-score) or who were identified as influential observations based on data exploration to ensure robustness of statistical analyses.

### Participant characteristics

We summarized participant characteristics using medians [interquartile ranges (IQRs)] and counts (proportions) for continuous and binary-categorical characteristics, respectively. We summarized HIV-specific clinical characteristics measured at both the POPPY baseline visit and at or near the POPPY-Sleep Sub-study visit when the plasma biomarkers were measured. We compared general baseline characteristics measured at the POPPY baseline visit between people with and without HIV using Wilcoxon rank-sum tests for continuous variables, and chi-squared/Fisher's exact tests for categorical variables, as appropriate. We also compared general baseline characteristics and HIV-specific clinical characteristics between the inflammatory plasma biomarker clusters using Kruskal–Wallis tests for continuous variables, and chi-squared/Fisher's exact tests for categorical variables, as appropriate.

### Linear regression analyses

Linear regression was used to estimate associations between the change in Global T-score and both HIV status and the specific subset of plasma biomarkers hypothesized to be associated with changes in cognitive function. Each of the plasma biomarkers was (natural) log-transformed and included as an independent variable in unadjusted and adjusted linear regression models separately to estimate the association of each plasma biomarker with the change in Global T-score. In adjusted analyses, we adjusted for important confounders identified *a priori*: age, sex, race, education and HIV status (for plasma biomarker analyses). We used likelihood ratio tests (LRTs) to first explore whether there were significant differences in changes in Global T-scores by HIV status before and after adjustment for important confounders. Subsequently, we used LRTs to explore whether there were significant associations between changes in Global T-scores and the specific subset of plasma biomarkers. We also explored whether any associations between changes in Global T-scores and the specific subset of plasma biomarkers varied by HIV status using LRTs if an interaction term between the log-transformed plasma biomarker and HIV status was statistically significant, after adjustment for important confounders. Average predicted Global T-score changes, and their 95% confidence intervals were generated from the final models across the observed range of biomarker concentrations that were statistically significant, holding other covariates constant at representative values.

All analyses were performed using R version 4.1.0, with two-sided *P*-values <0.05 considered to be statistically significant.

## Results

### Participant characteristics

Of the 465 participants who had reliable plasma biomarker measurements and were eligible for inclusion in analyses, 350 participants had complete data on Global T-scores collected at both the baseline and wave 3 visits. One participant was identified to have a particularly extreme change in Global T-scores and was excluded due to potentially inaccurate cognitive testing, further supported by their identification as an influential observation with a high degree of influence on regression estimates, resulting in a final sample of 349 participants.

Of participants in the sample, 254 (72.8%) were people with HIV, the median (IQR) age was 54 (50, 60) years and most were white (94.6%), male (85.1%), and men who have sex with men (MSM) (77.7%). Among those with HIV, most (249 (98.0%)) were on ART, 235 (93.3%) had HIV-RNA ≤50 copies/ml, and the median (IQR) CD4^+^ cell count was 627 (490, 792) cells/mm^3^ at the POPPY baseline visit (Table 1). Few changes in these HIV-specific measures were observed at the POPPY-Sleep Sub-study visit (Table 1). The 115 participants excluded because of missing data on change scores were more likely to be female, non-white, to have acquired HIV through sex between men and women, not consume alcohol currently and to have a BMI ≥30 kg/m^2^ (Table A1, Supplemental Digital Content). Compared to those included with HIV, those excluded with HIV had a lower median CD4^+^ T-cell count at both the POPPY baseline and Sleep Sub-study visits, shorter median time since HIV diagnosis and lower proportion with a history of dideoxynucleoside (d-drugs) use (Table A1, Supplemental Digital Content).

**Table 1:** Summary characteristics of participants included, overall and by people with and without HIV.

Median [IQR] or *n* (%)	Overall^a^ *N* = 349	People without HIV *N* = 95	People with HIV *N* = 254	*P*-value
(Socio-) demographics				
Age, years	54 (50, 60)	58 (54, 61)	53 (47, 59)	<0.001
Male	297 (85.1%)	66 (69.5%)	231 (90.9%)	<0.001
White	330 (94.6%)	91 (95.8%)	239 (94.1%)	0.53
Educational attainment – university degree or above	172 (50.3%)	47 (50.0%)	125 (50.4%)	0.95
Anthropometric measurements				
BMI ≥30 kg/m^2^	53 (15.3%)	16 (17.2%)	37 (14.6%)	0.55
Systolic blood pressure (mmHg)	127 (117, 138)	130 (118, 144)	126 (117, 137)	0.10
Diastolic blood pressure (mmHg)	79 (72, 85)	78 (72, 84)	79 (72, 86)	0.85
Lifestyle factors				
MSM sexuality/route of HIV acquisition	271 (77.7%)	56 (58.9%)	215 (84.6%)	<0.001
Current alcohol use^b^	299 (85.7%)	89 (93.7%)	210 (82.7%)	0.009
History of recreational drug use in past 6 months	83 (23.8%)	15 (15.8%)	68 (26.8%)	0.03
Ever injected drugs	28 (8.0%)	2 (2.1%)	26 (10.2%)	0.01
HIV-specific characteristics				
Measured at POPPY baseline visit				
HIV-RNA ≤50 copies/ml		–	235 (93.3%)	–
CD4^+^ T-cell count (cells/mm^3^)		–	627 (490, 792)	–
Nadir CD4^+^ T-cell count (cells/mm^3^)		–	199 (100, 320)	–
On any form of ART^c^		–	249 (98.0%)	–
History of dideoxynucleoside (d-drugs) use^d^		–	92 (36.2%)	–
History of any AIDS event		–	68 (26.8%)	–
Measured at or near POPPY-Sleep Sub-study visit				
HIV-RNA ≤50 copies/ml		–	238 (94.4%)	–
CD4^+^ T-cell count (cells/mm^3^)		–	660 (526, 864)	–
Nadir CD4^+^ T-cell count (cells/mm^3^)		–	190 (99, 306)	–
On any form of ART^c^		–	246 (96.9%)	–
On protease inhibitors		–	162 (63.8%)	–
On non-nucleoside reverse transcriptase inhibitors		–	196 (77.2%)	–
On integrase inhibitors		–	50 (19.7%)	–
Cumulative exposure to any form of ART, years^c^		–	10.8 (5.0, 16.9)	–
Years since HIV diagnosis		–	18.8 (11.3, 24.9)	–

ART, antiretroviral therapy; BMI, body mass index; IQR, interquartile range; MSM, men who have sex with men.

a The following variables have missing data (number of participants with missing data reported in parentheses): Educational attainment (7), BMI (3), baseline HIV-RNA ≤50 copies/ml (2), baseline CD4^+^ cell count (7), baseline nadir CD4^+^ cell count (13), POPPY-Sleep Sub-study visit HIV-RNA ≤50 copies/ml (2), POPPY-Sleep Sub-study visit CD4^+^ cell count (1), POPPY-Sleep Sub-study visit nadir CD4^+^ cell count (1), years since HIV diagnosis (at POPPY-Sleep Sub-study visit) (2).

b Current alcohol use is defined as any current alcohol use vs. no current alcohol use

c Note: Participants may have used more than one form of ART treatment.

d History of dideoxynucleoside (d-drugs) use includes previous use of didanosine (ddI), zalcitabine (ddC) and/or stavudine (d4T).

Among those included in the present analyses, people with and without HIV differed on several characteristics at baseline. People with HIV were younger than those without HIV (median age of 53 vs. 58 years, *P* < 0.001) as expected given the study design, were less likely to currently consume alcohol (82.7% vs. 93.7%, *P* = 0.009), and were more likely to be male (90.6% vs. 69.5%, *P* < 0.001), MSM (84.3% vs. 58.9%, *P* < 0.001), to have a history of recreational drug use in the 6 months prior to baseline (26.8% vs. 15.8%, *P* = 0.03) and to have ever injected drugs (10.2% vs. 2.1%, *P* = 0.01) (Table 1).

People with HIV had higher median concentrations of sCD14 [1744.0 vs. 1516.9 × 10^3^ picograms per millilitre (pg/ml), *P* < 0.001], sCD163 (679.3 vs. 474.8 × 10^3^ pg/ml, *P* < 0.001) and MCP-1 (157.8 vs. 140.9 pg/ml, *P* = 0.002). People with HIV were more likely to be in the ‘neurovascular’ cluster (45.7% vs. 34.7%) and less likely to be in the ‘gut/immune activation’ cluster (7.9% vs. 15.8%) compared to those without HIV (global *P*-value 0.04) (Table 2).

**Table 2:** Plasma biomarker concentrations (units in picograms per millilitre (pg/ml) unless otherwise stated), overall and by people with and without HIV.

Plasma biomarker/inflammatory plasma biomarker cluster	Overall (*N* = 349)	People without HIV (*N* = 95)	People with HIV (*N* = 254)	*P*-value
Neuronal injury				
NFL	57.5 (41.4, 77.9)	63.3 (45.4, 77.0)	55.7 (40.4, 77.9)	0.24
S100B	623.3 (514.6, 781.3)	666.3 (538.4, 790.3)	611.9 (506.9, 765.7)	0.11
Systemic inflammation				
IL-2	1.0 (0.6, 1.8)	0.9 (0.5, 2.7)	1.0 (0.6, 1.6)	0.49
IL-6	2.1 (1.5, 3.1)	2.1 (1.6, 3.3)	2.2 (1.5, 3.1)	0.88
TNF-α	5.1 (4.2, 6.6)	5.3 (4.0, 7.7)	5.0 (4.2, 6.4)	0.41
Innate immune activation				
sCD14 (×10^3^ pg/ml)	1622.1 (1335.0, 2036.3)	1516.9 (1239.4, 1825.0)	1744.0 (1398.3, 2117.5)	<0.001
sCD163 (×10^3^ pg/ml)	640.6 (407.8, 977.3)	474.8 (342.1, 804.9)	679.3 (430.7, 1012.2)	<0.001
IL-10	0.7 (0.5, 1.0)	0.8 (0.6, 1.2)	0.7 (0.5, 1.0)	0.16
MCP-1	152.7 (127.9, 194.1)	140.9 (119.6, 174.0)	157.8 (130.7, 202.9)	0.002
MIP-1α	524.8 (454.6, 597.0)	542.2 (454.7, 617.3)	524.1 (454.8, 592.4)	0.63
Inflammatory biomarker cluster				
‘Reference’	165 (47.3%)	47 (49.5%)	118 (46.5%)	0.04
‘Gut/immune activation’	35 (10.0%)	15 (15.8%)	20 (7.9%)	
‘Neurovascular’	149 (42.7%)	33 (34.7%)	116 (45.7%)	

### Global T-score changes: POPPY baseline and wave 3 study visits

The median (IQR) time between the baseline and wave 3 cognitive assessments was 26 (24, 29) months. Overall, Global T-scores increased from a mean of 47.7 (SD: 5.9) at baseline to 48.9 (5.4) at wave 3 (mean change 1.2 [95% CI 0.7, 1.7]) (Table 3); 133/349 (38.1%) participants experienced a decline in the Global T-Score between the two visits, but for most (111/133, 83.5% or 31.8% of the total group) this decline was <5 (a change that would be deemed to be clinically significant). The mean change in Global T-scores was 1.1 (0.6, 1.7) in people with HIV and 1.3 (0.5, 2.1) in people without HIV (*P* = 0.72 for difference between the two groups), with 98/254 (38.6%) and 35/95 (36.8%) of the two groups having a decline in Global T-scores between visits, respectively.

**Table 3:** Summary of Global T-Score measures assessed at baseline and wave 3 of the POPPY Study, overall and by people with and without HIV.

	Observed mean (standard deviation)			Estimated Mean difference (95% confidence interval (CI)) (reference: people without HIV)			
Outcome	Overall *N* = 349	People without HIV *N* = 95	People with HIV *N* = 254	Unadjusted		Adjusted^a^	
				Estimate (95% CI)	LRT *P*-value	Estimate (95% CI)	LRT *P*-value
Global T-Score – POPPY Study baseline visit	47.7 (5.9)	49.3 (4.8)	47.1 (6.1)	−2.2 (−3.6, −0.8)	0.002	−1.5 (−2.8, −0.1)	0.04
Global T-Score – POPPY Study wave 3 visit	48.9 (5.4)	50.7 (4.4)	48.3 (5.6)	−2.4 (−3.6, −1.1)	<0.001	−1.9 (−3.1, −0.6)	0.004
Change in Global T-Score (wave 3 – baseline)	1.2 (4.4)	1.3 (3.9)	1.1 (4.6)	−0.2 (−1.2, 0.8)	0.72	−0.4 (−1.5, 0.7)	0.48

LRT, likelihood ratio test.

a Adjusted for age, sex, education, and race.

Observed changes in the Global T-scores were stable across quintiles of the plasma biomarker concentrations overall but were slightly more variable by HIV status (Figures A1 and A2, Supplemental Digital Content). The estimated associations between plasma biomarkers and the change in Global T-scores between the two POPPY study visits are shown in Table 4. Higher concentrations of MIP-1α (−0.27 (−0.51, −0.03)) and sCD14 (−0.17 (−0.30, −0.03)) were associated with lower average increases in Global T-scores, though only the association with MIP−1α (−0.34 (−0.58, −0.10)) remained significant after adjustment. Plots of the average predicted change in Global T-score by values of MIP-1α and sCD14, overall and adjusted for demographic factors, are shown in Figure A3, Supplemental Digital Content. There was no evidence that the biomarker associations varied by HIV status (Table 4).

**Table 4:** Results of unadjusted and adjusted analyses exploring associations between Global T-score measures and specific plasma biomarkers.

		Unadjusted		Adjusted (age, sex, race and education and HIV status)		
Outcome	Plasma biomarker/index	Estimate (95% CI)*	LRT *P*-value	Estimate (95% CI)^a^	LRT *P*-value	LRT *P*-value (HIV status: plasma biomarker interaction)^b^
Change in Global T-Score (wave 3 – baseline)	S100B	0.02 (−0.10, 0.15)	0.71	0.02 (−0.11, 0.15)	0.78	0.28
	NFL	−0.03 (−0.11, 0.05)	0.46	−0.05 (−0.15, 0.04)	0.25	0.87
	IL-6	−0.03 (−0.11, 0.04)	0.37	−0.03 (−0.11, 0.05)	0.40	0.13
	IL-2	−0.04 (−0.08, 0.01)	0.09	−0.03 (−0.07, 0.02)	0.20	0.04
	TNF-α	−0.01 (−0.10, 0.09)	0.85	0.02 (−0.07, 0.12)	0.61	0.27
	sCD163	0.03 (−0.04, 0.10)	0.44	0.03 (−0.04, 0.10)	0.46	0.55
	MIP-1α	−0.27 (−0.51, −0.03)	0.02	−0.34 (−0.58, −0.10)	0.01	0.21
	MCP-1	−0.08 (−0.20, 0.04)	0.18	−0.09 (−0.21, 0.04)	0.17	0.56
	IL-10	0.01 (−0.07, 0.09)	0.86	0.03 (−0.05, 0.10)	0.53	0.92
	sCD14	−0.17 (−0.30, −0.03)	0.02	−0.13 (−0.27, 0.01)	0.06	0.34

LRT, likelihood ratio test.

a Note, interpret the coefficients for the biomarkers as the mean change in the Global T-score measure/outcome for every 10% increase in the biomarker concentration, for example, where a positive coefficient indicates “improved” cognitive function and a negative coefficient indicates “reduced” cognitive function for change scores.

b Testing for the addition of an interaction term between HIV status and the biomarker/index, while the adjusted estimate (95% CI) and *P*-value are from a model without an interaction term. [refer to Table A3, Supplemental Digital Content for interaction effects for people with and without HIV].

### Secondary analyses: inflammatory plasma biomarker clusters

Of the 349 participants included, 165 (47.3%) were in the ‘reference’ cluster, 35 (10.0%) in the ‘gut/immune activation’ cluster and the remaining 149 (42.7%) in the ‘neurovascular’ cluster (Table 2). Consistent with previous findings [28], there were statistically significant between-cluster differences for several baseline characteristics, with those in the ‘neurovascular’ cluster being more likely to be living with HIV or obesity, those in the ‘gut/Immune activation’ cluster having a higher median age and systolic blood pressure, and those in the ‘reference’ cluster being more likely to have a university education (Table A2, Supplemental Digital Content).

While a slight increase was observed in the Global T-scores between the POPPY baseline and wave 3 visits as indicated by the positive mean change scores (95% CI) of within-individual change (1.2 (−0.5, 3.0), 1.0 (0.2, 1.7), 1.4 (0.8, 2.0) for ‘gut/immune activation’, ‘neurovascular’ and ‘reference’ clusters, respectively), there were no statistically significant between-cluster differences before or after adjustment for important confounders (Table 5). These data were consistently compatible with models without an interaction term between the inflammatory plasma biomarker cluster and HIV status variables relative to models with this interaction term, suggesting no evidence that between-cluster differences varied across people with and without HIV.

**Table 5:** Summary of regression analyses of Global T-Score measures assessed at baseline and wave 3 of the POPPY Study by inflammatory biomarker cluster.

					Estimated mean difference (95% confidence interval)						
	Observed mean (standard deviation)				Unadjusted (reference cluster: ‘reference’)			Adjusted (reference cluster: ‘reference’)^a^			
	Overall *N* = 349	‘Reference’ *N* = 165	‘Gut/immune activation’ *N* = 35	‘Neurovascular’ *N* = 149	‘Gut/immune activation’ *N* = 35	‘Neurovascular’ *N* = 149	LRT *P*-value	‘Gut/immune activation’ *N* = 35	‘Neurovascular’ *N* = 149	LRT p-value	LRT *P*-value – interaction HIV status-cluster^b^
Change in Global T-Score (wave 3 – baseline)	1.2 (4.4)	1.4 (4.1)	1.2 (5.0)	1.0 (4.5)	−0.2 (−1.8, 1.4)	−0.4 (−1.4, 0.5)	0.67	0.4 (−1.3, 2)	−0.6 (−1.6, 0.4)	0.33	0.96

LRT, likelihood ratio test.

a Adjusted for HIV status, age, sex, education, and race.

b Testing for the addition of an interaction term between HIV status and the inflammatory biomarker cluster variable, while the “LRT *P*-value adjusted” is from a model without an interaction term.

## Discussion

Over a 2-year period, we observed a mild mean increase in Global T-scores in our cohort of people with and without HIV. While we did not find clear associations between the change in Global T-scores and most biomarkers, we found that higher concentrations of MIP-1α and sCD14 were associated with lower average increases in Global T-scores, consistent with literature suggesting a link between chemokine dysregulation and cognitive impairment. Chemokines such as MIP-1α have been implicated in neuroinflammation and blood–brain barrier dysfunction, and elevated concentrations have been observed both in people with HIV on ART [31,32] and individuals without HIV with cognitive impairment [33]. However, after adjustment for key demographic covariates, we found that only the MIP-1α association remained statistically significant, and there was no evidence that these relationships differed by HIV status. This further suggests that the impact of specific inflammatory pathways on cognitive function is likely multifactorial and relevant to brain health in all, rather than being unique to HIV. These findings contribute to the existing evidence base on the associations between biomarkers of systemic inflammation and innate immune activation with changes in cognitive function [34,35].

Beyond the specific biomarker associations with changes in cognitive function, our findings demonstrated a distinct inflammatory profile in participants with HIV who, despite being virologically suppressed on effective ART, exhibited significantly higher concentrations of certain inflammatory plasma biomarkers, particularly sCD14, sCD163, and MCP-1, suggesting ongoing immune activation. These results are consistent with previous reports indicating that people with HIV on virologically suppressive ART continue to exhibit elevated biomarkers of systemic inflammation and monocyte activation including sCD14 and sCD163 compared to cohorts without HIV [7,36,37]. Elevated concentrations of sCD14 and sCD163 have also been independently associated with cognitive impairment in people with HIV, underscoring their clinical relevance [38,39].

Building on the observations of distinct HIV-associated inflammatory profiles, participants with HIV were more likely to be classified in the ‘neurovascular’ inflammatory cluster and less likely in the ‘gut/immune activation’ cluster. This finding may indicate distinctive inflammatory phenotypes in people with HIV, potentially shaped by chronic viral infection, ART exposure, immune dysregulation, and other comorbidities and conditions associated with inflammation [40–42] that disproportionately impact people with HIV.

In contrast to studies that have identified associations between immune activation profiles and cognitive trajectories in people with HIV [43], in our study we observed that the mean Global T-score change was broadly similar, with no statistically significant observed differences between the inflammatory clusters, either before or after adjustment, and differences did not vary by HIV status. Several factors likely contribute to these divergent findings. Participants in POPPY were generally healthier, older, predominantly white men with well controlled HIV and few advanced comorbidities, whereas the Women's Interagency HIV Study (WIHS) cohort, as presented in the publication by Rubin *et al.* [43], included only women, greater racial diversity, and more individuals exposed to structural disadvantage, all of which may amplify inflammation–cognition relationships. Furthermore, the WIHS analysis captured inflammatory signatures closer to ART initiation and evaluated cognition over a longer follow-up, whereas POPPY assessed immune markers and cognitive change within a relatively stable, virologically suppressed population over a shorter period. It is also notable that participants excluded from our analysis, who were younger, more often female or from non-white ethnic groups, and with a higher comorbidity burden, more closely resembled the WIHS cohort, where stronger inflammation–cognition associations have previously been reported. Overall, these differences suggest that cognitive trajectories in ageing populations, with or without HIV, are influenced by a complex interplay of demographic, biological, psychosocial, and structural factors, and that inflammation-related cognitive risk is not specific to HIV alone.

Our data suggests that while distinctive inflammatory profiles exist (as represented by the inflammatory clusters in this instance), these inflammatory patterns were not strongly predictive of short-term cognitive function change in our cohort of people with well controlled HIV and preserved immune function. The absence of associations at the cluster level, despite modest biomarker-specific effects, may reflect dilution of MIP-1α and sCD14 signals when combined with other markers, potentially obscuring associations within broader inflammatory clusters.

The pattern of modest improvements alongside subtle cognitive fluctuations in our cohort, particularly among people with HIV, aligns with findings from other longitudinal studies in cohorts of older people with HIV who are virologically suppressed on ART and without advanced immunosuppression [44]. Whilst a proportion of participants did experience a decline in Global T-scores, these changes were generally small, did not exceed the threshold typically considered clinically significant in most, and were similar regardless of HIV status, suggesting that they were largely a reflection of random variation. The comparable changes in Global T-score between people with and without HIV suggest that HIV status alone may not fully explain individual differences in cognitive trajectories, a conclusion echoed by studies in both populations that have highlighted the multifactorial role of comorbidities, substance use, and psychosocial factors in shaping cognitive outcomes [45,46].

Our study has several strengths, including a well characterized, longitudinally followed cohort, the inclusion of people with HIV and demographically similar people without HIV, and detailed immune phenotyping using inflammatory biomarker clustering. However, some limitations should be acknowledged. First, the observational design of our study limits our ability to draw causal inferences. Second, the cohort was predominantly white, male, and MSM, which limits the generalizability of our findings to other key populations affected by HIV, such as women and people of non-white ethnicities. Inflammation-cognition relationships may differ in women and people of non-white ethnicities due to biological differences in immune responses, sex-specific hormonal influences, and differential exposure to social and structural determinants of health [47,48]. Additionally, individuals with different HIV acquisition routes may experience distinct comorbidity profiles or patterns of immune activation, which could influence cognitive outcomes [49]. These factors highlight the need for studies in more diverse populations to fully understand the interplay between inflammation and cognitive function in people living with HIV. In addition, individuals with HIV differed from those not living with HIV in several sociodemographic and behavioural characteristics. Individuals with HIV were younger and more likely to be white, male, MSM, have a history of recreational or injecting drug use and were less likely to consume alcohol, reflective of the demographic profile of the overall POPPY study cohort [26]. These differences may reflect broader social and behavioural patterns in the epidemiology of people with HIV. While certain factors such as comorbidities and psychosocial variables may influence biomarker changes, these factors themselves are often affected by HIV and adjusting for them as potential confounders might inadvertently obscure meaningful associations between HIV, biomarkers, and cognitive function. For this reason, these variables were not included as covariates in our analyses, and this should be borne in mind when interpreting the results. Finally, while statistically significant, the absolute changes in Global T-scores over the 2-year period were small and may not reflect clinically meaningful relevance.

Future research should aim to validate these findings in larger populations of people with HIV and demographically similar people without HIV, and to replicate these findings in other representative populations, including women, older adults, and ethnically diverse populations. Integrating more detailed cognitive testing with longitudinal neuroimaging data and advanced immune profiling may uncover the biological mechanisms linking chronic inflammation and cognition outcomes. Understanding these pathways in both people with and without HIV and integrating inflammatory biomarker profiles into predictive models of cognitive decline could offer insights into identifying at-risk individuals and developing targeted interventions.

## Conclusions

In conclusion, while our findings underscore persistent immune activation in people on virologically suppressive ART and suggest modest associations between certain inflammatory biomarkers and cognitive function over a 2-year period, we also highlight the intricacy of these relationships. The apparent lack of interaction between HIV status and inflammatory clusters on changes in cognitive function further supports the view that other non-HIV factors likely also play important roles in determining cognitive trajectories over time. Our results highlight the complexity of factors that may influence inflammation-related cognitive impairment in the context of people on ART with virological suppression, confirming that further research into the interplay between chronic inflammation and cognitive outcomes is warranted, particularly in people ageing with HIV.

## Acknowledgements

POPPY Management Team: Marta Boffito, Patrick Mallon, Frank Post, Caroline Sabin, Memory Sachikonye, Alan Winston, Christina Prechtl. POPPY Scientific Steering Committee: Jane Anderson, David Asboe, Marta Boffito, Lucy Garvey, Patrick Mallon, Frank Post, Jasmini Alagaratnam, Anton Pozniak, Caroline Sabin, Memory Sachikonye, Jaime Vera, Ian Williams, Alan Winston. POPPY Sites and Trials Unit: Caldecot Centre, King's College Hospital (Frank Post, Lucy Campbell, Selin Yurdakul, Sara Okumu, Louise Pollard, Beatriz Santana Suárez, Luella Hanbury) Department of Infection and Population Health, UCL (Ian Williams, Damilola Otiko, Laura Phillips, Rosanna Laverick, Michelle Beynon, Anna-Lena Salz, Abigail Severn, Michelle Beynon, Gosala Gopalakrishnan) Clinical Research Facility Allan at St Mary's, Brighton and Sussex University Hospital (Martin Fisher, Amanda Clarke, Jaime Vera, Andrew Bexley, Celia Richardson, Sarah Kirk, Rebecca Gleig, Leigh Greenland) HIV Molecular Research Group, School of Medicine, UCD (Patrick Mallon, Alan Macken, Bijan Ghavani-Kia, Joanne Maher, Maria Byrne, Ailbhe Flaherty, Aoife McDermott, Riya Negi, Alejandro Garcia-Leon and Aoife Cotter) Homerton Sexual Health Services, Homerton University Hospital (Jane Anderson, Sifiso Mguni, Rebecca Clark, Rhiannon Nevin-Dolan, Sambasivarao Pelluri, Tracey Fong) Ian Charleson Day Centre, Royal Free Hospital (Margaret Johnson, Nnenna Ngwu, Nargis Hemat, Anne Carroll, Sabine Kinloch, Mike Youle, Sara Madge and Katie Spears) Imperial Clinical Trials Unit, Imperial College London (Daphne Babalis, Jodi Meyerowitz, Christina Prechtl) St. Mary's Hospital London, Imperial College Healthcare NHS Trust (Alan Winston, Lucy Garvey, Merle Henderson, Claire Peterson, Wilbert Ayap, Allan Lisenco and Ian McGuinness) St Stephen's Centre, Chelsea and Westminster Hospital (Marta Boffito, David Asboe, Anton Pozniak, Margherita Bracchi, Jasmini Alagaratnam, Nicole Pagani, Maddalena Cerrone, Daniel Bradshaw, Francesca Ferretti, Chris Higgs, Elisha Seah, Stephen Fletcher, Michelle Anthonipillai, Ashley Moyes, Katie Deats, Irtiza Syed, Clive Matthews, Peter Fernando, Cherry Colcol). POPPY methodology/statistics: Caroline Sabin, Hajra Okhai, Luxsena Sukumaran. NIHR HPRU Steering Committee: Professor C.A. Sabin (HPRU Director), Dr J. Saunders (UKHSA Lead), Professor C. Mercer, Dr H. Mohammed, Professor G. Rait, Dr R. Simmons, Professor W. Rosenberg, Dr T. Mbisa, Professor R. Raine, Dr S. Mandal, Dr R. Yu, Dr S. Ijaz, Dr F. Lorencatto, Dr R. Hunter, Dr K. Foster and Dr M. Tahir.

Sources of support and funding: The POPPY-Sleep sub-study (which these analyses used sleep data collected from) was funded by National Heart Lung and Blood Institute (R01 HL131049). The parent POPPY Study waves 1 to 3 was funded by investigator-initiated grants from BMS, Gilead Sciences, Janssen, Merck and ViiV Healthcare (EudraCT Number: 2012- 003581–40; Sponsor Protocol Number: CRO1992). The POPPY study waves 4 and 5 is funded by investigator-initiated grants from Gilead Sciences, ViiV Healthcare and Merck Sharp & Dohme (UK) Limited (Sponsor protocol number: 19SM5112). The study is also supported by the National Institute for Health Research (NIHR) Biomedical Research Centre based at Imperial College Healthcare NHS Trust and Imperial College London and by an NIHR Senior Investigator Award to Professor C.A. Sabin. This material is also the result of work supported with resources and the use of facilities at the Minneapolis Veterans Affairs Medical Center, Minneapolis, USA and the Boise VA Medical Center, Idaho, USA.

The views expressed in this article are those of the authors and do not necessarily reflect the position or policy of the Department of Veterans Affairs or the United States government, the NHS, the NIHR, the Department of Health or the funders.

**Author contributions:** Conceptualization: N.B., A.W., C.S.

Data curation and validation: N.B. and C.S.

Contributed to the design of the data analysis: N.B., J.A., A.W. and C.S.

Performed the data analysis: N.B. and C.S.

Funding acquisition: C.S. and A.W.

Supervision: C.S. and A.W.

Wrote the first draft of the manuscript: N.B. and J.A.

All authors contributed to the review and editing of the final submitted manuscript for intellectual content.

### Conflicts of interest

J.A. has received research grants from Gilead Sciences, National Institute for Health and Care Research (NIHR) Imperial Biomedical Research Centre (BRC) and British HIV Association (BHIVA), paid to her institution, and has received speaker's fees and/or advisory board honoraria and/or conference travel support from Gilead Sciences Ltd, MSD and ViiV Healthcare, unrelated to this work.

P.W.G.M. has received honoraria, speaker fees and/or conference travel support from Gilead Sciences Ltd, MSD and Janssen Cilag.

F.B. has received institutional grants from Gilead Sciences Ltd., MSD and ViiV Healthcare, and speakers’ fees from Gilead Sciences Ltd.

L.W. has received speaker/advisory fees from ViiV, MSD, Janssen and AbbVie. She is an investigator on trials sponsored by Gilead, ViiV and MSD.

F.A.P. has received institutional grants from Gilead Sciences Ltd, ViiV, MSD and Immunocore. He has received honoraria from Gilead Sciences Ltd.

J.V. has received speakers’ fees and research funding from Gilead Sciences Ltd., MSD, ViiV Healthcare and Piramal Imaging.

A.W. has received honoraria or research grants on behalf of Imperial College London or been a consultant or investigator in clinical trials sponsored by Bristol-Myers Squibb, Gilead Sciences Ltd, GlaxoSmithKline, Janssen-Cilag, Roche and ViiV Healthcare. AW is supported in part by the NIHR Biomedical Research Centre of Imperial College NHS Trust.

C.S. has received funding for the provision of educational material, membership of speaker panels and advisory boards for Gilead Sciences Ltd, ViiV Healthcare and MSD.

The remaining authors declared no conflicts of interest related to this manuscript.

## Supplementary Material

Supplemental Digital Content

## References

[1] Rasmussen LD May MT Kronborg G Larsen CS Pedersen C Gerstoft J. Time trends for risk of severe age-related diseases in individuals with and without HIV infection in Denmark: a nationwide population-based cohort study. *Lancet HIV* 2015; 2:e288–e298. 26423253 10.1016/S2352-3018(15)00077-6

[2] Guaraldi G Orlando G Zona S Menozzi M Carli F Garlassi E. Premature age-related comorbidities among HIV-infected persons compared with the general population. *Clin Infect Dis* 2011; 53:1120–1126. 21998278 10.1093/cid/cir627

[3] Schouten J Wit FW Stolte IG Kootstra NA Van Der Valk M Geerlings SE. Cross-sectional comparison of the prevalence of age-associated comorbidities and their risk factors between hiv-infected and uninfected individuals: the age H IV cohort study. *Clin Infect Dis* 2014; 59:1787–1797. 25182245 10.1093/cid/ciu701

[4] Chenciner L Barber TJ. Noninfective complications for people living with HIV. *Med (United Kingdom)* 2022; 50:304–307. 10.1016/j.mpmed.2022.02.012 PMC8964336 35368724

[5] Zicari S Sessa L Cotugno N Ruggiero A Morrocchi E Concato C. Immune activation, inflammation, and non-AIDS co-morbidities in HIV-infected patients under long-term ART. *Viruses* 2019; 11:200. 30818749 10.3390/v11030200 PMC6466530

[6] Deeks SG. HIV infection, inflammation, immunosenescence, and aging. *Annu Rev Med* 2011; 62:141–155. 21090961 10.1146/annurev-med-042909-093756 PMC3759035

[7] Hunt PW Lee SA Siedner MJ. Immunologic biomarkers, morbidity, and mortality in treated HIV infection. *J Infect Dis* 2016; 214:S44–S50. 27625430 10.1093/infdis/jiw275 PMC5021241

[8] Castillo-Mancilla JR Brown TT Palella FJ Macatangay BJC Breen EC Jacobson LP. Partial normalization of biomarkers of inflammation and immune activation among virally suppressed men with HIV infection and high ART adherence. *Open Forum Infect Dis* 2020; 7:ofaa099. 32322603 10.1093/ofid/ofaa099 PMC7162619

[9] Neuhaus J Jacobs DR Baker JV Calmy A Duprez D Rosa A La. Markers of inflammation, coagulation, and renal function are elevated in adults with HIV infection. *J Infect Dis* 2010; 201:1788–1795. 20446848 10.1086/652749 PMC2872049

[10] Heaton RK Clifford DB Franklin DR Woods SP Ake C Vaida F. HIV-associated neurocognitive disorders persist in the era of potent antiretroviral therapy: Charter Study. *Neurology* 2010; 75:2087–2096. 21135382 10.1212/WNL.0b013e318200d727 PMC2995535

[11] Simioni S Cavassini M Annoni JM Rimbault Abraham A Bourquin I Schiffer V. Cognitive dysfunction in HIV patients despite long-standing suppression of viremia. *AIDS* 2010; 24:1243–1250. 19996937 10.1097/QAD.0b013e3283354a7b

[12] del Palacio M Álvarez S Muñoz-Fernández ÁA. HIV-1 infection and neurocognitive impairment in the current era. *Rev Med Virol* 2012; 22:33–45. 21990255 10.1002/rmv.711

[13] Brew BJ Letendre SL. Biomarkers of HIV related central nervous system disease. *Int Rev Psychiatry* 2008; 20: doi:10.1080/09540260701878082. 10.1080/09540260701878082 18240064

[14] Everall I Vaida F Khanlou N Lazzaretto D Achim C Letendre S. Cliniconeuropathologic correlates of human immunodeficiency virus in the era of antiretroviral therapy. *J Neurovirol* 2009; 15:360–370. 20175693 10.3109/13550280903131915 PMC3078805

[15] Harezlak J Buchthal S Taylor M Schifitto G Zhong J Daar E. Persistence of HIV-associated cognitive impairment, inflammation, and neuronal injury in era of highly active antiretroviral treatment. *AIDS* 2011; 25:625–633. 21297425 10.1097/QAD.0b013e3283427da7 PMC4326227

[16] Robertson K Liner J Meeker RB. Antiretroviral neurotoxicity. *J Neurovirol* 2012; 18:388–399. 22811264 10.1007/s13365-012-0120-3 PMC3581315

[17] Gongvatana A Harezlak J Buchthal S Daar E Schifitto G Campbell T. Progressive cerebral injury in the setting of chronic HIV infection and antiretroviral therapy. *J Neurovirol* 2013; 19:209–218. 23613008 10.1007/s13365-013-0162-1 PMC3740160

[18] Cohen RA Seider TR Navia B. HIV effects on age-associated neurocognitive dysfunction: premature cognitive aging or neurodegenerative disease?. *Alzheimers Res Ther* 2015; 7:37. 25848401 10.1186/s13195-015-0123-4 PMC4386102

[19] Wallace DR. HIV-associated neurotoxicity and cognitive decline: therapeutic implications. *Pharmacol Ther* 2022; 234:108047. 34848202 10.1016/j.pharmthera.2021.108047

[20] Brew BJ Pemberton L Cunningham P Law MG. Levels of human immunodeficiency virus type 1 RNA in cerebrospinal fluid correlate with AIDS dementia stage. *J Infect Dis* 1997; 175:963–966. 9086160 10.1086/514001

[21] Ellis RJ Hsia K Spector SA Nelson JA Heaton RK Wallace MR. Cerebrospinal fluid human immunodeficiency virus type 1 RNA levels are elevated in neurocognitively impaired individuals with acquired immunodeficiency syndrome. *Ann Neurol* 1997; 42:679–688. 9392566 10.1002/ana.410420503

[22] McArthur JC McClernon DR Cronin MF Nance-Sproson TE Saah AJ St Clair M. Relationship between human immunodeficiency virus-associated dementia and viral load in cerebrospinal fluid and brain. *Ann Neurol* 1997; 42:689–698. 9392567 10.1002/ana.410420504

[23] Ryan LA Zheng J Brester M Bohac D Hahn F Anderson J. Plasma levels of soluble CD14 and tumor necrosis factor-( type II receptor correlate with cognitive dysfunction during human immunodeficiency virus type 1 infection. *J Infect Dis* 2001; 184:699–706. 11517430 10.1086/323036

[24] Gartner S Liu Y. Insights into the role of immune activation in HIV neuropathogenesis. *J Neurovirol* 2002; 8: 10.1080/13550280290049525 11935459

[25] Sacktor N McDermott MP Marder K Schifitto G Selnes OA McArthur JC. HIV-associated cognitive impairment before and after the advent of combination therapy. *J Neurovirol* 2002; 8:136–142. 11935465 10.1080/13550280290049615

[26] Bagkeris E Burgess L Mallon PW Post FA Boffito M Sachikonye M. Cohort profile: the pharmacokinetic and clinical observations in PeoPle over fiftY (POPPY) study. *Int J Epidemiol* 2018; 47:1391–1392e. 29746638 10.1093/ije/dyy072

[27] Kunisaki KM De Francesco D Sabin CA Winston A Mallon PWG Anderson J. Sleep disorders in human immunodeficiency virus: a substudy of the Pharmacokinetics and Clinical Observations in People over Fifty (POPPY) study. *Open Forum Infect Dis* 2021; 8:ofaa561. 33447632 10.1093/ofid/ofaa561 PMC7781463

[28] Bakewell N Sabin CA Negi R Garcia-Leon A Winston A Sachikonye M. Biomarker associations with insomnia and secondary sleep outcomes in persons with and without HIV in the POPPY-Sleep substudy: a cohort study. *Sleep* 2022; 45:zsac212. 36104003 10.1093/sleep/zsac212 PMC9742892

[29] De Francesco D Underwood J Post FA Vera JH Williams I Boffito M. Defining cognitive impairment in people-living-with-HIV: the POPPY study. *BMC Infect Dis* 2016; 16:617. 27793128 10.1186/s12879-016-1970-8 PMC5084371

[30] De Francesco D Underwood J Anderson J Boffito M Post FA Sachikonye M. Correlation between computerised and standard cognitive testing in people with HIV and HIV-negative individuals. *AIDS Care* 2021; 33:1296–1307. 33356492 10.1080/09540121.2020.1865518 PMC9448413

[31] Kroll KW Woolley G Terry K Premeaux TA Shikuma CM Corley MJ. Multiplex analysis of cytokines and chemokines in persons aging with or without HIV. *AIDS Res Hum Retroviruses* 2023; 39:367–380. 37097212 10.1089/aid.2022.0183 PMC11074629

[32] Spagnolo-Allende A Schnall R Liu M Igwe KC Laing KK Chesebro AG. Serum inflammation markers associated with altered brain white matter microstructure in people with HIV on antiretroviral treatment. *Neurol Sci* 2023; 44:2159–2166. 36710283 10.1007/s10072-023-06613-2 PMC10635284

[33] Mekhora C Lamport DJ Spencer JPE. An overview of the relationship between inflammation and cognitive function in humans, molecular pathways and the impact of nutraceuticals. *Neurochem Int* 2024; 181:105900. 39522696 10.1016/j.neuint.2024.105900

[34] Farina MP Kim JK Hayward MD Crimmins EM. Links between inflammation and immune functioning with cognitive status among older Americans in the Health and Retirement Study. *Brain Behav Immun Health* 2022; 26:100559. 36439057 10.1016/j.bbih.2022.100559 PMC9694056

[35] Fang Y Doyle MF Chen J Alosco ML Mez J Satizabal CL. Association between inflammatory biomarkers and cognitive aging. *PLoS One* 2022; 17:e0274350. 36083988 10.1371/journal.pone.0274350 PMC9462682

[36] Tenorio AR Zheng Y Bosch RJ Krishnan S Rodriguez B Hunt PW. Soluble markers of inflammation and coagulation but not T-cell activation predict non-AIDS-defining morbid events during suppressive antiretroviral treatment. *J Infect Dis* 2014; 210:1248–1259. 24795473 10.1093/infdis/jiu254 PMC4192039

[37] Spooner RK Taylor BK Moshfegh CM Ahmad IM Dyball KN Emanuel K. Neuroinflammatory profiles regulated by the redox environment predicted cognitive dysfunction in people living with HIV: a cross-sectional study. *EBioMedicine* 2021; 70:103487. 34280780 10.1016/j.ebiom.2021.103487 PMC8318860

[38] Burdo TH Weiffenbach A Woods SP Letendre S Ellis RJ Williams KC. Elevated sCD163 in plasma but not cerebrospinal fluid is a marker of neurocognitive impairment in HIV infection. *AIDS* 2013; 27:1387–1395. 23435298 10.1097/QAD.0b013e32836010bd PMC3844286

[39] Lyons JL Uno H Ancuta P Kamat A Moore DJ Singer EJ. Plasma sCD14 is a biomarker associated with impaired neurocognitive test performance in attention and learning domains in HIV infection. *J Acquir Immune Defic Syndr* 2011; 57:371–379. 21646912 10.1097/QAI.0b013e3182237e54 PMC3159710

[40] Osimo EF Pillinger T Rodriguez IM Khandaker GM Pariante CM Howes OD. Inflammatory markers in depression: a meta-analysis of mean differences and variability in 5166 patients and 5083 controls. *Brain Behav Immun* 2020; 87: doi:10.1016/j.bbi.2020.02.010. 10.1016/j.bbi.2020.02.010 PMC7327519 32113908

[41] Cuevas AG Ong AD Carvalho K Ho T Chan SW Allen JD. Discrimination and systemic inflammation: a critical review and synthesis. *Brain Behav Immun* 2020; 89:465–479. 32688027 10.1016/j.bbi.2020.07.017 PMC8362502

[42] Matthews T Rasmussen LJH Ambler A Danese A Eugen-Olsen J Fancourt D. Social isolation, loneliness, and inflammation: a multicohort investigation in early and mid-adulthood. *Brain Behav Immun* 2024; 115:727–736. 37992788 10.1016/j.bbi.2023.11.022 PMC11194667

[43] Rubin LH Xu Y Norris PJ Wang X Dastgheyb R Fitzgerald KC. Early inflammatory signatures predict subsequent cognition in long-term virally suppressed women with HIV. *Front Integr Neurosci* 2020; 14:20. 32390808 10.3389/fnint.2020.00020 PMC7193823

[44] Wang Y Liu M Lu Q Farrell M Lappin JM Shi J. Global prevalence and burden of HIV-associated neurocognitive disorder: a meta-analysis. *Neurology* 2020; 95:e2610–e2621. 32887786 10.1212/WNL.0000000000010752

[45] Hsu HC Bai CH. Individual and environmental factors associated with cognitive function in older people: a longitudinal multilevel analysis. *BMC Geriatr* 2022; 22:243. 35321640 10.1186/s12877-022-02940-9 PMC8941778

[46] Ellis RJ Marquine MJ Kaul M Fields JA Schlachetzki JCM. Mechanisms underlying HIV-associated cognitive impairment and emerging therapies for its management. *Nat Rev Neurol* 2023; 19:668–687. 37816937 10.1038/s41582-023-00879-y PMC11052664

[47] Hanamsagar R Bilbo SD. Sex differences in neurodevelopmental and neurodegenerative disorders: focus on microglial function and neuroinflammation during development. *J Steroid Biochem Mol Biol* 2016; 160:127–133. 26435451 10.1016/j.jsbmb.2015.09.039 PMC4829467

[48] Davidson-Turner KJ Farina MP Hayward MD. Racial/Ethnic differences in inflammation levels among older adults 56+: an examination of sociodemographic differences across inflammation measure. *Biodemography Soc Biol* 2024; 69:75–89. 38807566 10.1080/19485565.2024.2356672 PMC11257156

[49] Piggott DA Mehta SH Rubin LH Sun J Leng SX Kirk GD. Cognitive function and mortality among persons aging with HIV and injection drug use. *AIDS* 2025; 39:1214–1223. 40053483 10.1097/QAD.0000000000004169 PMC12383706

