# Supplementary material for: Associations between plasma biomarkers and changes in cognitive function over two years in people with and without HIV
Source: AIDS. 2026 Jan 14;40(5):611–21. doi: 10.1097/QAD.0000000000004435 (PMC13034748; doi:10.1097/QAD.0000000000004435)
Supplement: Supplemental Digital Content [file aids-40-611-s001.docx]

**Supplementary Tables and Figures**

| **Table A1.** Summary characteristics of participants by Excluded and Included based on having data on primary outcome of the change score Global T-scores between POPPY Study baseline and wave 3 visits | | | | |
| --- | --- | --- | --- | --- |
| Median [IQR] or n (%) | Overall*  N=464 | Included  N=349 | Excluded  N=115 | p-value |
| (Socio-)Demographics |  |  |  |  |
| Age, years | 54 (50, 60) | 54 (50, 60) | 55 (50, 60) | 0.92 |
| Living with HIV | 342 (73.7%) | 254 (72.8%) | 88 (76.5%) | 0.43 |
| Male | 374 (80.6%) | 297 (85.1%) | 77 (67.0%) | <0.001 |
| White | 408 (87.9%) | 330 (94.6%) | 78 (67.8%) | <0.001 |
| Educational attainment - University degree or above | 217 (48.2%) | 172 (50.3%) | 45 (41.7%) | 0.12 |
| Anthropometric Measurements |  |  |  |  |
| BMI ≥30 kg/m^2^ | 81 (17.6%) | 53 (15.3%) | 28 (24.6%) | 0.03 |
| Systolic Blood Pressure (mmHg) | 126 (117, 140) | 127 (117, 138) | 126 (117, 140) | 0.84 |
| Diastolic Blood Pressure (mmHg) | 79 (72, 86) | 79 (72, 85) | 79 (69, 87) | 0.89 |
| Lifestyle Factors |  |  |  |  |
| MSM sexuality/route of HIV transmission | 332 (71.6%) | 271 (77.7%) | 61 (53.0%) | <0.001 |
| Current alcohol use** | 383 (82.5%) | 299 (85.7%) | 84 (73.0%) | 0.002 |
| History of recreational drug use in past 6 months | 107 (23.1%) | 83 (23.8%) | 24 (20.9%) | 0.52 |
| Ever injected drugs | 32 (6.9%) | 28 (8.0%) | 4 (3.5%) | 0.10 |
| HIV-specific characteristics |  |  |  |  |
| Measured at POPPY Baseline Visit |  |  |  |  |
| HIV-RNA <50 copies/mL | 313 (92.1%) | 235 (93.3%) | 78 (88.6%) | 0.17 |
| CD4+ T-Cell Count (cells/mm^3^) | 610 (470, 780) | 627 (490, 792) | 570 (427, 737) | 0.02 |
| Nadir CD4+ T-Cell Count (cells/mm^3^) | 200 (99, 306) | 199 (100, 320) | 200 (82, 276) | 0.59 |
| On any form of ART*** | 334 (97.7%) | 249 (98.0%) | 85 (96.6%) | 0.43 |
| History of dideoxynucleoside (d-drugs) use**** | 110 (32.2%) | 92 (36.2%) | 18 (20.5%) | 0.006 |
| History of any AIDS event | 95 (27.8%) | 68 (26.8%) | 27 (30.7%) | 0.48 |
| Measured at or Near POPPY-Sleep Sub-study Visit |  |  |  |  |
| HIV-RNA <50 copies/mL | 320 (94.1%) | 238 (94.4%) | 82 (93.2%) | 0.67 |
| CD4+ T-Cell Count (cells/mm^3^) | 630 (486, 841) | 660 (526, 864) | 546 (432, 802) | 0.002 |
| Nadir CD4+ T-Cell Count (cells/mm^3^) | 194 (87, 290) | 190 (100, 306) | 196 (71, 270) | 0.41 |
| On any form of ART*** | 332 (97.1%) | 246 (96.9%) | 86 (97.7%) | >0.99 |
| On Protease Inhibitors | 211 (61.7%) | 162 (63.8%) | 49 (55.7%) | 0.18 |
| On Non-Nucleoside Reverse Transcriptase Inhibitors | 331 (96.8%) | 245 (96.5%) | 86 (97.7%) | 0.74 |
| On Integrase Inhibitors | 62 (18.1%) | 50 (19.7%) | 12 (13.6%) | 0.20 |
| Cumulative exposure to any form of ART, years*** | 9.9 (4.5, 16.7) | 10.8 (5.0, 16.9) | 8.0 (3.9, 14.2) | 0.07 |
| Years since HIV Diagnosis | 17.4 (10.9, 24.4) | 18.8 (11.3, 24.9) | 14.8 (9.4, 21.2) | 0.02 |
| *Overall participant count is based on total number of participants with reliable biomarker data who were eligible for inclusion. The following variables have missing data (number of participants missing data reported in parentheses): University degree or above (14), Obese (4), Systolic Blood Pressure (3), Diastolic Blood Pressure (3), Ever injected drugs (1), baseline HIV-RNA <50 copies/mL (2), baseline CD4+ Cell Count (12), baseline Nadir CD4+ Cell Count (17), POPPY-Sleep visit HIV-RNA <50 copies/mL (2), POPPY-Sleep Sub-study visit CD4+ Cell Count (1), POPPY-Sleep Sub-study visit Nadir CD4+ Cell Count (1), Years since HIV Diagnosis (at POPPY-Sleep Sub-study visit) (4).  ** Current alcohol use is defined as any current alcohol use versus no current alcohol use  ***Note: Participants may have used more than one form of ART treatment.  ****History of dideoxynucleoside (d-drugs) use includes previous use of didanosine (ddI), zalcitabine (ddC) and/or stavudine (d4T)  Abbreviations: IQR = interquartile range; BMI = body mass index; MSM = men who have sex with men; ART = antiretroviral therapy | | | | |

| **Table A2.** Summary characteristics of participants included, overall and by inflammatory biomarker cluster | | | | | |
| --- | --- | --- | --- | --- | --- |
| Median [IQR] or n (%) | Overall*  N=349 | ‘Reference’  N=165 | ‘Gut/immune activation’  N=35 | ‘Neurovascular’  N=149 | p-value |
| (Socio-)Demographics | | | | | |
| Age, years | 54 (50, 60) | 53 (48, 60) | 57 (52, 61) | 55 (51, 60) | 0.04 |
| Living with HIV | 254 (72.8%) | 118 (71.5%) | 20 (57.1%) | 116 (77.9%) | 0.04 |
| Male | 297 (85.1%) | 140 (84.8%) | 27 (77.1%) | 130 (87.2%) | 0.32 |
| White | 330 (94.6%) | 158 (95.8%) | 32 (91.4%) | 140 (94.0%) | 0.51 |
| Educational attainment - University degree or above | 172 (50.3%) | 96 (59.3%) | 14 (42.4%) | 62 (42.2%) | 0.01 |
| Anthropometric Measurements | | | | | |
| BMI ≥30 kg/m^2^ | 53 (15.3%) | 14 (8.5%) | 5 (14.3%) | 34 (23.1%) | 0.002 |
| Systolic Blood Pressure (mmHg) | 127 (117, 138) | 126 (116, 136) | 134 (125, 152) | 128 (117, 140) | 0.03 |
| Diastolic Blood Pressure (mmHg) | 79 (72, 85) | 78 (72, 84) | 82 (72, 89) | 80 (72, 86) | 0.39 |
| Lifestyle Factors | | | | | |
| MSM sexuality/route of HIV transmission | 271 (77.7%) | 128 (77.6%) | 26 (74.3%) | 117 (78.5%) | 0.86 |
| Current alcohol use** | 299 (85.7%) | 141 (85.5%) | 31 (88.6%) | 127 (85.2%) | 0.87 |
| History of recreational drug use in past 6 months | 83 (23.8%) | 36 (21.8%) | 6 (17.1%) | 41 (27.5%) | 0.31 |
| Ever injected drugs | 28 (8.0%) | 14 (8.5%) | 2 (5.7%) | 12 (8.1%) | 0.96 |
| HIV-specific characteristics | | | | | |
| Measured at POPPY Baseline Visit |  |  |  |  |  |
| HIV-RNA <50 copies/mL | 235 (93.3%) | 111 (94.9%) | 17 (89.5%) | 107 (92.2%) | 0.42 |
| CD4+ T-Cell Count (cells/mm^3^) | 627 (490, 792) | 618 (510, 785) | 651 (437, 700) | 630 (480, 818) | 0.61 |
| Nadir CD4+ T-Cell Count (cells/mm^3^) | 199 (100, 320) | 222 (114, 324) | 160 (114, 329) | 160 (80, 297) | 0.10 |
| On any form of ART*** | 249 (98.0%) | 115 (97.5%) | 20 (100.0%) | 114 (98.3%) | >0.99 |
| History of dideoxynucleoside (d-drugs) use**** | 92 (36.2%) | 38 (32.2%) | 6 (30.0%) | 48 (41.4%) | 0.29 |
| History of any AIDS event | 68 (26.8%) | 25 (21.2%) | 8 (40.0%) | 35 (30.2%) | 0.11 |
| Measured at or Near POPPY-Sleep Sub-study Visit |  |  |  |  |  |
| HIV-RNA <50 copies/mL | 238 (94.4%) | 113 (96.6%) | 16 (84.2%) | 109 (94.0%) | 0.07 |
| CD4+ T-Cell Count (cells/mm^3^) | 660 (526, 864) | 666 (520, 834) | 571 (459, 682) | 692 (538, 918) | 0.23 |
| Nadir CD4+ T-Cell Count (cells/mm^3^) | 190 (100, 306) | 217 (105, 305) | 160 (119, 365) | 166 (80, 302) | 0.28 |
| On any form of ART*** | 246 (96.9%) | 113 (95.8%) | 19 (95.0%) | 114 (98.3%) | 0.43 |
| On Protease Inhibitors | 162 (63.8%) | 76 (64.4%) | 12 (60.0%) | 74 (63.8%) | 0.93 |
| On Non-Nucleoside Reverse Transcriptase Inhibitors | 196 (77.2%) | 87 (73.7%) | 13 (65.0%) | 96 (82.8%) | 0.09 |
| On Integrase Inhibitors | 50 (19.7%) | 18 (15.3%) | 6 (30.0%) | 26 (22.4%) | 0.17 |
| Cumulative exposure to any form of ART, years*** | 10.8 (5.0, 16.9) | 9.1 (4.5, 16.3) | 9.7 (3.6, 15.6) | 12.8 (5.6, 17.4) | 0.22 |
| Years since HIV Diagnosis | 18.8 (11.3, 24.9) | 17.3 (11.3, 24.6) | 16.2 (12.2, 22.8) | 20.4 (11.2, 25.9) | 0.37 |
| *The following variables have missing data (number of participants missing data reported in parentheses): University degree or above (7), BMI ≥30 kg/m^2^ (3), baseline HIV-RNA <50 copies/mL (2), baseline CD4+ Cell Count (7), baseline Nadir CD4+ Cell Count (13), POPPY-Sleep visit HIV-RNA <50 copies/mL (2), POPPY-Sleep Sub-study visit CD4+ Cell Count (1), POPPY-Sleep Sub-study visit Nadir CD4+ Cell Count (1), Years since HIV Diagnosis (at POPPY-Sleep Sub-study visit) (2).  ** Current alcohol use is defined as any current alcohol use versus no current alcohol use  ***Note: Participants may have used more than one form of ART treatment.  ****History of dideoxynucleoside (d-drugs) use includes previous use of didanosine (ddI), zalcitabine (ddC) and/or stavudine (d4T)  Abbreviations: IQR = interquartile range; BMI = body mass index; MSM = men who have sex with men; ART = antiretroviral therapy | | | | | |

| **Table A3.** Estimates (95% confidence interval (CI)) of the main exposure (i.e., specific plasma biomarkers) and HIV-exposure interaction terms and Likelihood ratio test p-value for an interaction (as presented in **Table 4** of the main manuscript) (all models adjusted for HIV status, age, sex, race and education) | | | | | |
| --- | --- | --- | --- | --- | --- |
| Global T-score Measure/Outcome | Plasma Biomarker/Index | Main Exposure Term Estimate  (95% CI) | Exposure:HIV Interaction Term Estimate  (95% CI) | Combined Estimate for People with HIV  (95% CI) | LRT p-value (interaction term)* |
| Global T-Score –  Change score (wave 3 – baseline) | S100B | -0.07 (-0.30, 0.14) | 0.15 (-0.12, 0.42) | 0.07 (-0.09, 0.23) | 0.28 |
|  | NFL | -0.07 (-0.27, 0.13) | 0.02 (-0.21, 0.24) | -0.05 (-0.15, 0.05) | 0.87 |
|  | IL-6 | 0.07 (-0.09, 0.23) | -0.14 (-0.32, 0.05) | -0.07 (-0.16, 0.02) | 0.13 |
|  | IL-2 | 0.04 (-0.04, 0.11) | -0.09 (-0.19, 0.00) | -0.06 (-0.11, -0.01) | 0.04 |
|  | TNF-⍺ | 0.10 (-0.07, 0.27) | -0.12 (-0.32, 0.09) | -0.01 (-0.13, 0.11) | 0.27 |
|  | sCD163 | -0.01 (-0.14, 0.12) | 0.05 (-0.11, 0.20) | 0.04 (-0.04, 0.12) | 0.55 |
|  | MIP-1⍺ | -0.09 (-0.54, 0.36) | -0.34 (-0.87, 0.19) | -0.43 (-0.71, -0.15) | 0.21 |
|  | MCP-1 | -0.15 (-0.39, 0.10) | 0.08 (-0.20, 0.37) | -0.06 (-0.21, 0.08) | 0.56 |
|  | IL-10 | 0.03 (-0.12, 0.19) | -0.01 (-0.19, 0.17) | 0.02 (-0.07, 0.12) | 0.92 |
|  | sCD14 | -0.03 (-0.29, 0.24) | -0.15 (-0.46, 0.16) | -0.18 (-0.34, -0.01) | 0.34 |
| *Note, interpret the coefficients for the biomarkers as the mean change in the Global T-score measure/outcome for every 10% increase in the biomarker concentration (i.e., semi-elasticities), for example, where a positive coefficient indicates “improved” cognitive function and a negative coefficient indicates “reduced” cognitive function for change scores. Also note that the LRT p-value does not pertain to the estimate (95% CI) presented for people with HIV, it is from a LRT comparing regression model without and with an interaction. The combined effect presented for people with HIV is testing a linear combination, or joint, hypothesis test of the sum of the main and interaction terms equal to 0. Therefore, it is possible that the 95% CI presented for the estimate specific to people with HIV to be statistically significant (from a joint effect size of 0), while the LRT is not statistically significant. | | | | | |


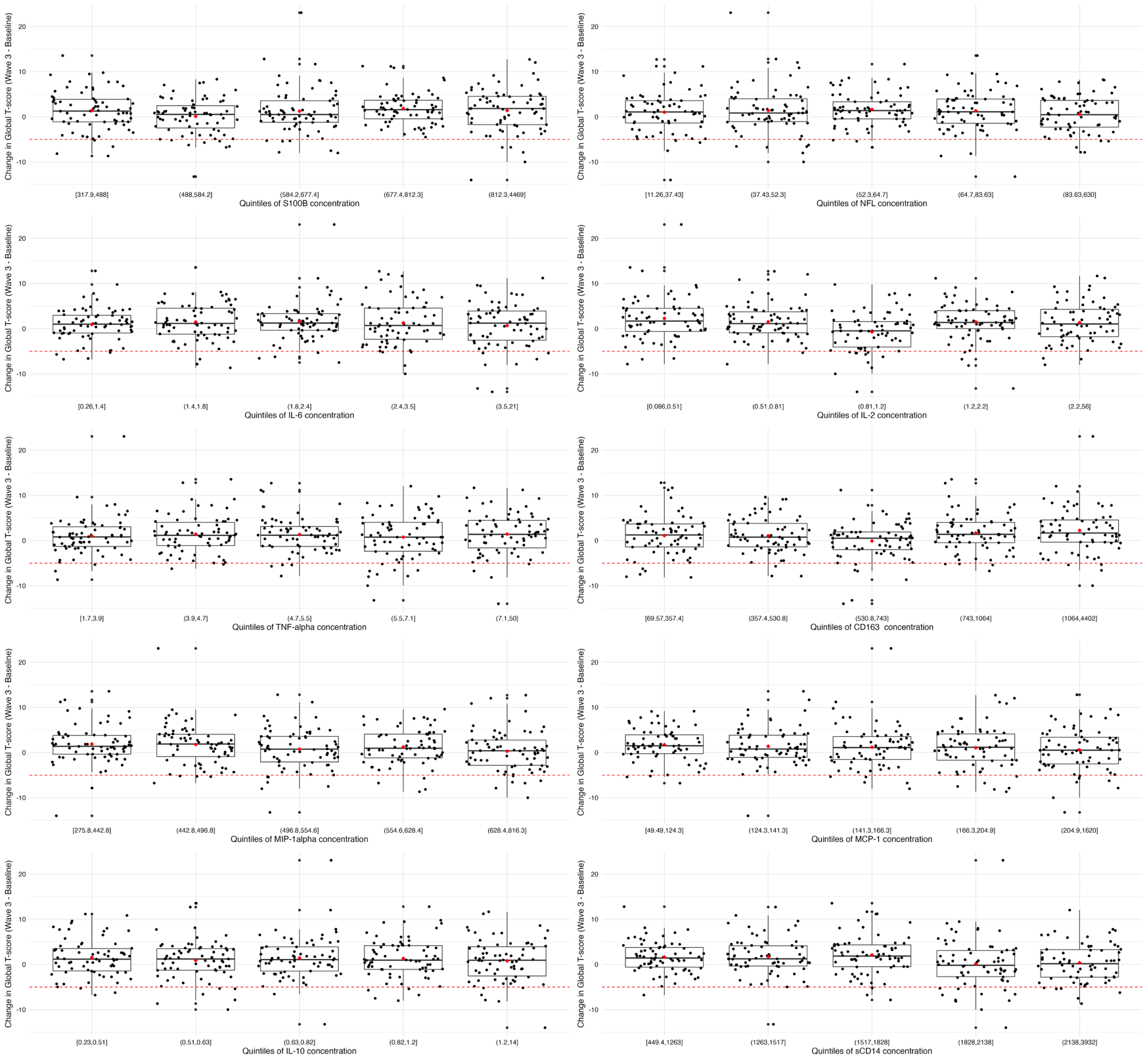
**Figure A1.** Boxplots of Change in Global T-score (Wave 3-Baseline) by Quintile of Biomarker Concentration (jittered black dots are participants’ observed changes; red dot is the mean; red line indicates a decline of 5, which below this line is considered a clinically significant decline)

**Figure A2.** Boxplots of Change in Global T-score (Wave 3- Baseline) by Quintile of Biomarker Concentration AND Living with HIV Status (jittered black dots are participants’ observed changes; green dot is the mean; red line indicates a decline of 5, which below this line is considered a clinically significant decline)


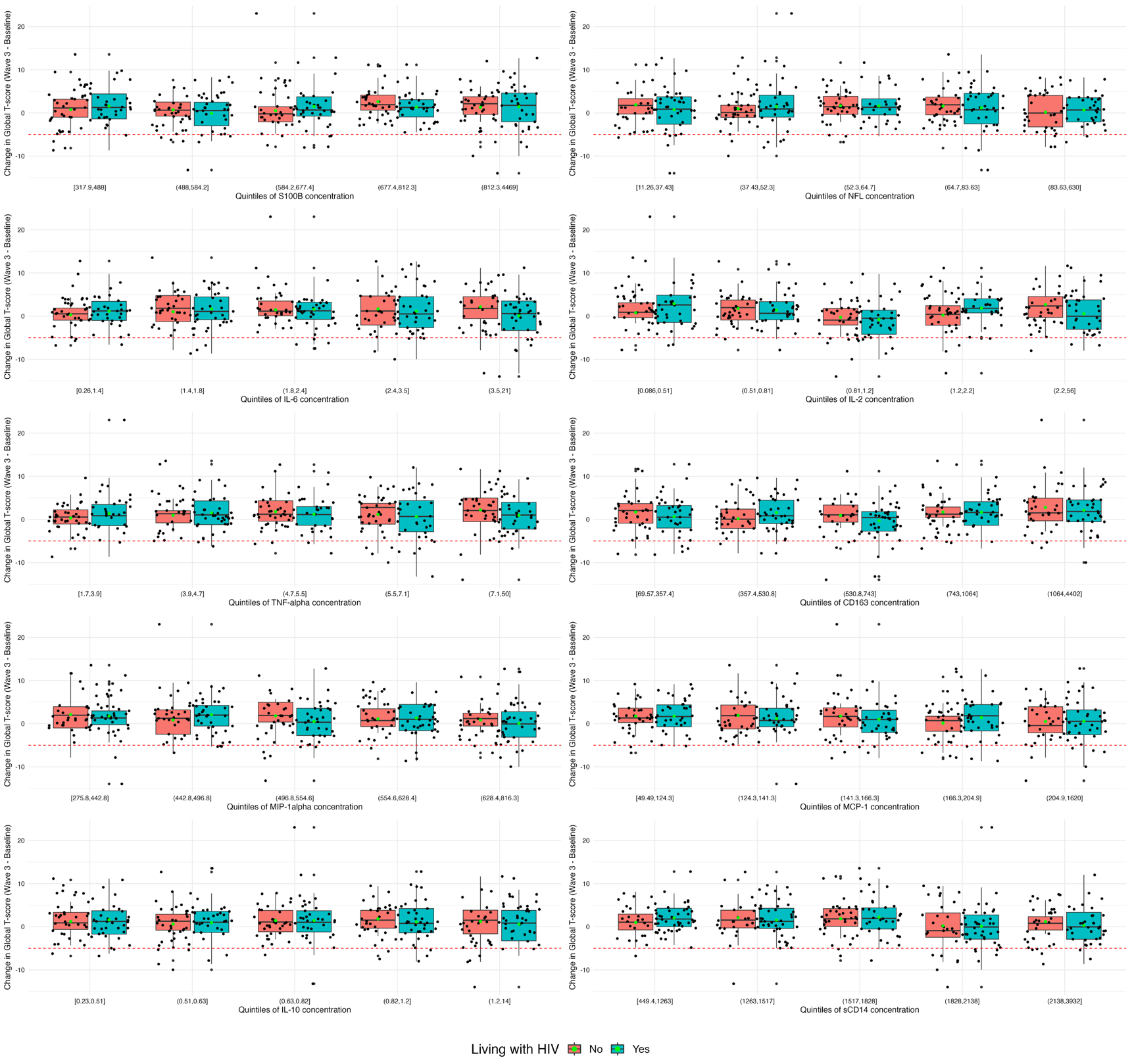


**Note:** Biomarker concentrations for sCD163 and sCD14 are in x103 picograms per millilitre; all other biomarker concentrations are in picograms per millilitre.

**Figure A3.** Average predicted change in Global T-score by selected biomarker concentrations (and 95% confidence intervals): unadjusted and adjusted


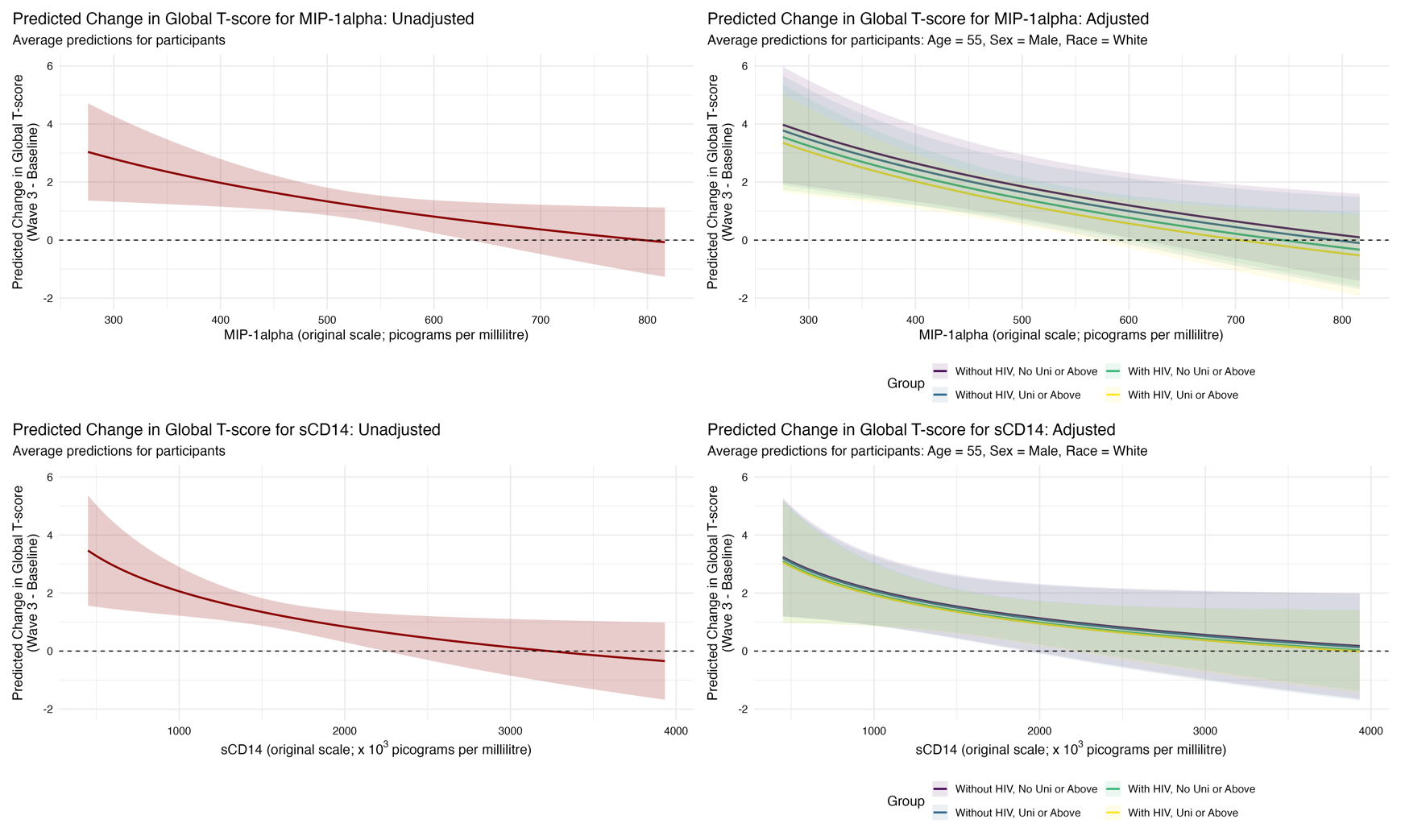


**Note:** Adjusted average predictions are presented for a male participant, aged 55 years, of White race. Age 55 was chosen as it is the closest multiple of five to the median age of the study population, and male and White race represent the most common sex and race categories within the study. Adjusted average predictions are presented by people living with and without HIV and educational attainment, as the grouping by HIV status is clinically important, and educational attainment represents a key demographic factor with a near 50/50 split in the study population.
